# PGPR strain *Paenibacillus polymyxa* SQR-21 potentially benefits watermelon growth by re-shaping root protein expression

**DOI:** 10.1186/s13568-017-0403-4

**Published:** 2017-05-25

**Authors:** Yaoyao E, Jun Yuan, Fang Yang, Lei Wang, Jinghua Ma, Jing Li, Xiaowei Pu, Waseem Raza, Qiwei Huang, Qirong Shen

**Affiliations:** Jiangsu Provincial Key Lab of Organic Solid Waste Utilization and Jiangsu Collaborative Innovation Center for Organic Solid Waste Utilization, and National Engineering Research Center for Organic-based Fertilizer, Nanjing Agricultural University, Nanjing, 210095 China

**Keywords:** *Paenibacillus polymyxa*, Proteomics, Hydroponic split-roots, Biological control

## Abstract

*Paenibacillus polymyxa* (SQR-21) is not only a plant growth-promoting rhizobacteria, but also an effective biocontrol agent against *Fusarium* wilt disease of watermelon. For the better understanding and clarifying the potential mechanisms of SQR-21 to improve watermelon growth and disease resistance, a split-root methodology in hydroponic and LC–MS technology with the label free method was used to analyze the key root proteins involved in watermelon metabolism and disease resistance after the inoculation of SQR-21. Out of 623 identified proteins, 119 proteins were differentially expressed when treatment (SQR-21 inoculation) and control (no bacterial inoculation) were compared. Among those, 57 and 62 proteins were up-regulated and down-regulated, respectively. These differentially expressed proteins were identified to be involved in signal transduction (ADP-ribosylation factor, phospholipase D), transport (aspartate amino-transferase), carbohydratemetabolic (glucose-6-phosphate dehydrogenase, UDP-glucose pyrophosphorylase), defense and response to stress (glutathione S-transferase, Ubiquitin-activating enzyme E1), and oxidation–reduction process (thioredoxin peroxidase, ascorbate peroxidase). The results of this study indicated that SQR-21 inoculation on the watermelon roots benefits plant by inducing the expression of several proteins involved in growth, photosynthesis, and other metabolic and physiological activities.

**Electronic supplementary material:**

The online version of this article (doi:10.1186/s13568-017-0403-4) contains supplementary material, which is available to authorized users.

## Introduction

Plant growth promoting rhizobacteria (PGPR), live freely in the soil and plant rhizosphere, can promote plant growth and mineral nutrition and restrain harmful microbes (Kloepper and Schroth 1978). PGPRs can enhance plant growth by a variety of mechanisms like phosphate solubilization (Vessey 2003; Kim et al. 1998), siderophore production (Pinton et al. 2008), nitrogen fixation (Zakria et al. 2008), 1-aminocyclopropane-1-carboxylate (ACC) deaminase production (Adams and Yang 1979), phytohormone production (Belimov et al. 2005), induction in systemic resistance (Raupach et al. 1996), production of volatile organic compounds (VOCs) (Ryu et al. 2003), promoting beneficial plant–microbe symbioses, quorum sensing (QS) signal interference and biofilm formation etc. (Bhattacharyya and Jha 2012). Among the thousands of reported PGPRs, Paenibacillus polymyxa (formerly Bacillus polymyxa) is one of most famous PGPR used as effective biocontrol agent (Raza et al. 2008). The mechanisms of disease resistance and prevention can be simply summarized as direct effects and indirect effects. Direct effects are mainly through the secretion of antibiotics, hydrolytic enzymes and other substances directly inhibiting or killing the pathogenic microorganisms (Tenover 2006). Indirect effects have two pathways: firstly, stimulate the production of plant disease related proteins to induce plant disease resistance; secondly, secrete plant growth promoting hormones and activate the nutrient availability in the rhizosphere (Avis et al. 2008).

Plant-bacteria interactions are widely described; symbiotic organisms interact intimately with plants and provide them with metabolic advantages (Herder and Parniske 2009; Hou et al. 2009). Researchers showed that *P. polymyxa* strains effectively colonize the rhizosphere and plant roots and prevent various plant diseases caused by fungi, bacteria, and nematodes (Timmusk et al. 2009). *P. polymyxa* GBR-1 inhibited the root knot nematode egg hatching, poison larvae, and effectively reduced the tomato root knot formation (Khan et al. 2008). *P. polymyxa* HKA-15 metabolites effectively prevented the citrus canker (Mageshwaran et al. 2011). In addition, *P. polymyxa* strains help plant absorb nutrients, solubilize phosphorus and produce plant growth regulating hormones (indole-acetic acid, cytokinins, etc.) (Spaepen et al. 2007; Lal and Tabacchioni 2009). Petersen et al. found that co-inoculation of *P. polymyxa* and soybean Rhizobium significantly improved the colonization of soybean Rhizobium, thus promoted the nitrogen fixation (Petersen et al. 1996). *P. polymyxa* secrete phytic acid enzyme which can remove the hexakisphosphate group of inositol to dissolve phosphate. Besides, this enzyme also can combine the important mineral nutrients (Zn^2+^, Fe^2+^ and Ca^2+^) to eliminate chelate formation of phytic acid salt (Kerovuo et al. 1998).

Proteomics is the large-scale study of proteins, particularly their structures and functions. The use of differential proteomics applied to botany and crop science to study the relevant molecular mechanisms is increasing, which explained the mechanisms of crop genetic expression and response to the environment at the molecular level (Xu et al. 2008). Kim et al. used 2-DE technology to study the changes of expressed proteins in rice leaves at 24, 48, and 72 h after infection by *Pyricularia grisea* (Kim et al. 2003). Jones et al. infected *Pseudomonas syringae* to *Arabidopsis* leaves and measured the changes of proteins at 1.5-6 h after bacterial inoculation. This study successfully identified expression of 52 proteins changed significantly after bacterial inoculation (Jones et al. 2006). Mahmood et al. analyzed the cytoplasmic and membrane proteins from rice leaves pre-treated with *Xanthomonas* by 2-DE and MS, and identified 20 proteins which was significantly changed in abundance when faced with bacterial inoculation (Mahmood et al. 2006). *P. polymyxa* SQR-21 (SQR-21) isolated by the Jiangsu Provincial Key Lab of Organic Solid Waste Utilization, China was found to be a very effective biocontrol agent (Ling et al. 2010; Wu et al. 2008). Evidence supported that some organic acids from watermelon root exudates could induce the root colonization of SQR-21, such as malic acid and citric acid (Ling et al. 2011). The strain SQR-21, used for the preparation of biological fertilizer could significantly reduce the population of *Fusarium oxysporum* in the watermelon wilt diseased soil. Both in the pot and in field experiments, SQR-21 was showed excellent pathogen antagonistic and plant growth promotion effects. There is no information that how the watermelon root protein expression is changed after the colonized by a PGPR strain SQR-21. In order to detect the root protein expression when colonized by SQR-21 and evaluate the potential functions of the proteins which were differentially expressed, an adopt label free method and LC–MS technology were used to analyze the root expressed proteins during the interaction of watermelon and SQR-21. A hydroponics split-root system was designed to verify the alterations in the expression of proteins in SQR-21-inoculated and un-inoculated watermelon roots. This study provides insights into the communication between plants and PGPR, which may be helpful to promote the application of SQR-21 in the future agricultural production.

## Materials and methods

### Bacteria culture

The SQR-21 (CGMCC accession no: 1544; China General Microbiology Culture Collection Center) was isolated and identified by the Provincial Key Lab of Organic Solid Waste Utilization (Jiangsu, China) (Zhang et al. 2008). The culture sample (1 mL) of SQR-21 and the *gfp*-tagged strain SQR-21-*gfp* (Cao et al. 2011) were incubated in 100 mL liquid nutrition broth (3 g beef extract, 10 g peptone, 5 g NaCl and 1 L water, pH 7.2–7.4, 115 °C for 30 min sterilization) in a shaker at 30 °C and 170 rpm for 2 days. The cells were obtained by centrifugation (6000 rpm at 4 °C for 8 min), washed twice with sterile water and re-suspended in 10 mL sterile water. The concentration of bacterial suspension (1 × 10^9^ cfu/mL) was determined by the serial dilution plate counting method (Qiu et al. 2014).

### Plant materials

Watermelon seeds (Zaojia 84–24, obtained from the Jiangsu Academy of Agricultural Sciences) were surface-sterilized with household bleach (1% NaClO) for 15 min, then rinsed four times with sterile water. The seeds were placed in growth chamber at 30 °C covered with wet filter paper during the sprouting process. For germination, when seed shell cracked, and exposed burgeen, then selected seeds germinated in same degree were sown in moist sterilized mixture of perlite and vermiculite (V:V = 1:1). After 15 days, the seedlings (two true leaves unfolded) were gently transplanted into split-roots box containing modified Hoagland’s plant nutrition solution, KNO_3_ 5 mM/L, Ca(NO_3_)_2_ 2 mM/L, MgSO_4_ 2 mM/L, Fe-EDTA 0.05 mM/L, phosphate buffer (KH2PO4–K2HPO4, pH 5, 1 mM/L), microelement [(100×), H_3_BO_3_ 0.434 g, MnSO_4_·H_2_O 1.762 6 g, CuSO_4_·5H_2_O 0.0798 g, ZnSO_4_·7H_2_O 0.172 g, NaCl 0.585 g, CoCl_2_·6H_2_O 0.00129 g in 1 L]. The seedings of watermelons were transplanted to inoculation with SQR-21 at two-true-leaves stage. The nutrient solution was refreshed every 5 days.

### Experimental design

**Fig. 1:**
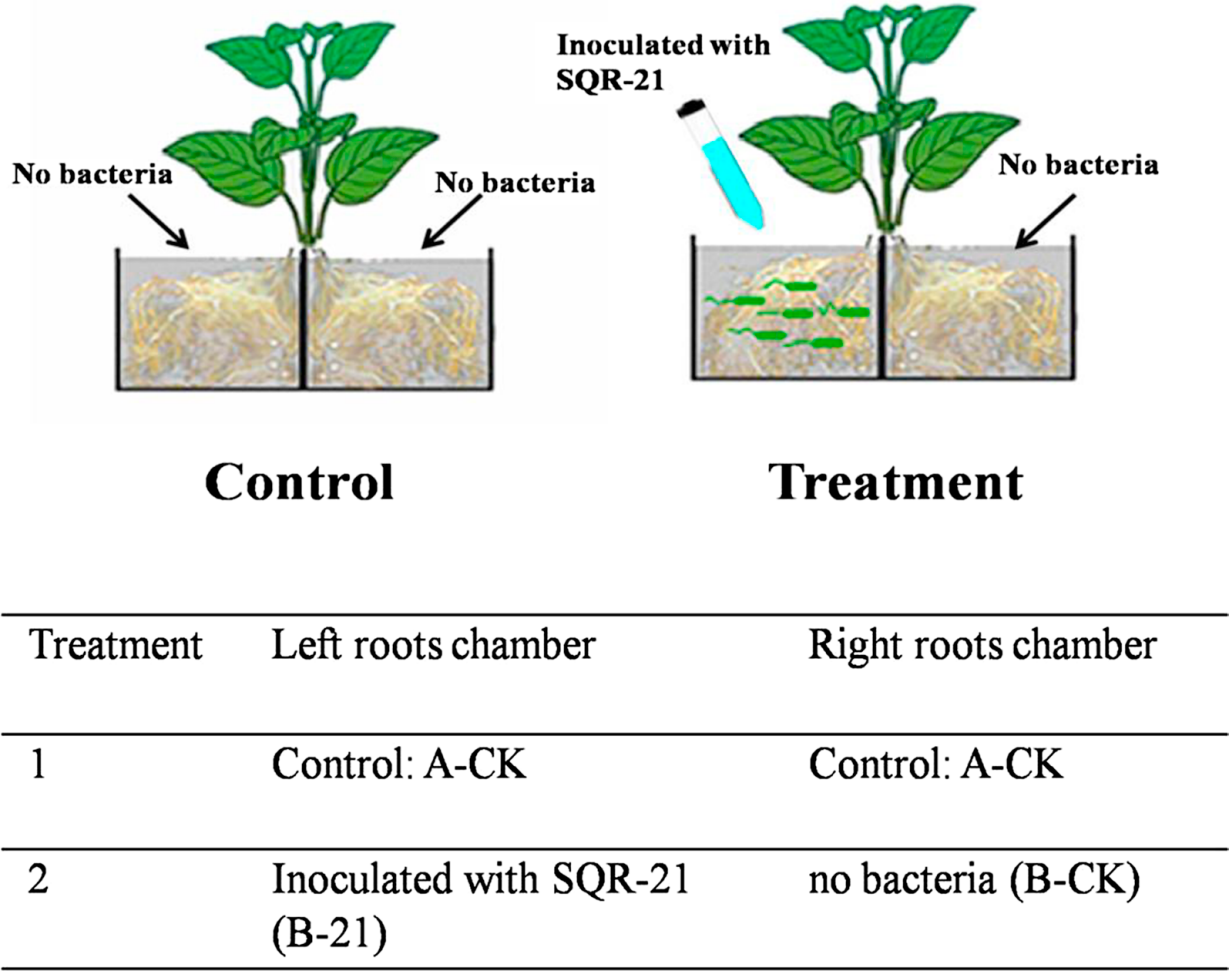
Schematic representation of the experimental design with the hydroponic split-roots system, which allows SQR-21 and watermelon roots co-cultured together in one half box, and the remaining half roots at the other half box were not contaminated by the strain

Root proteins collection assay was carried out using a hydroponic split-roots system. The split-root compartment system consisted of two compartments which were glued together but separated by a glass plate. Firstly, the watermelon with roots growth good were chosen, based on the number of lateral roots, trying to make each side of root number equal, and then planted in the two split-root compartments. The glass plate separating such two split-root compartments was impermeable for roots and microbes (Fig. 1). The following two treatments were designed: (1) control: no inoculation in both sides of the system was A-CK; (2) treatment: one side of roots inoculated with SQR-21 was B-21, and the root on the other side without any inoculation was B-CK. In this study, to avoid the effects of colonized bacteria, we only collected the samples from B-CK and A-CK. The concentration of inoculated SQR-21 was 1 × 10^6^ cfu/mL based on the serial dilution plate counting method. Each treatment included three replicates, and each replicates was three plants in one split-root box. All the plants were placed in a greenhouse with the photo-period of 16 h light (30 °C)/8 h dark (26 °C). Every 5 days, the nutrient solution was replaced for plant growth as well as SQR-21 bacterial cells. After the incubation of 30 days, the plants were harvested for root protein extraction and analysis.

### Root colonization confirmation

To determine whether the SQR-21 strain successfully colonized the watermelon roots, the suspension of SQR-21-*gfp* (1 × 10^6^ cfu/mL) was used to colonize the sterile watermelon seedlings in Hoagland’s medium. The root samples collected at 1, 3, and 5 days after the bacteria suspension first inoculated, then cut fresh roots into 1–2 cm long pieces, washed with sterile distilled water, placed on micro-slides, and directly observed by a confocal laser scanning microscope (CLSM) (Leica Model TCS SP2, Heidelberg, Germany). Images were obtained using Leica confocal software (version 2.61). To quantify the SQR-21 cells attached to the roots, the root samples were ground in a small mortar grand and quantified by the dilution plate counting method.

### Collection of roots and extraction of roots proteins

For the root collection, roots from three plants of one same split-root box were pooled as one sample. Firstly, plants were removed from split-root boxes and washed roots gently with the ultrapure water twice. Then the roots were cut with sterilized scissors and dried out by filter paper, next snap frozen directly in liquid nitrogen and stored at −80 °C for use. The total root proteins were extracted using plant protein extraction kit (Kangweishiji CW Bio, Beijing, China) according to the manufacturer’s instruction. Firstly, working-liquors were prepared according to volume of protease inhibitor cocktail: plant protein extraction reagent ratio of 1:99 (plant protein extraction reagent needed pre-cool). Then, adding the working-liquors, based on sample: working-liquors ratio of 1:5 (w:v) in a centrifugal tube. Next, ice-bath 20 min, and centrifuged at 4 °C 12,000 rpm for 20 min. Finally, collected supernatant to new centrifugal tube and stored at −80 °C for use. The protein extraction was quantified using BCA protein quantification kit (Dingguochangsheng Biotech Co. Ltd. Beijing, China).

### Protein digestion

The protein (100 μg) was digested using FASP (Filtered aide sample preparation) method with Trypsin Gold (Promega, Madison, WI, USA). First of all, the protein sample was mixed with 200 μL urea aqueous solution buffer (UA buffer) containing 5 μL 5 mM DTT Tris (8 M urea, 50 mM—HCl pH 8.0) in a 10 kDa ultra-centrifuge tube and reduced for 1.5 h at room temperature in dark. After reduction, the solution was centrifuged at 14,000*g* for 15 min, the flow-through was discarded and added 200 μL UA buffer and 5 μL 25 mM IAM, vortexed (600 rpm) for 1 min. Later, the solution was kept at room temperature in the dark for 30 min and centrifuged to abandon the filtrate. After that, 200 μL 50 mM NH_4_HCO_3_ solution (pH = 7.8) was added and centrifuged at 14,000*g* for 15 min, discarded the flow-through repeated this step for twice. The enzyme trypsin was added at the protein: trypsin ratio of 50:1 (v:v) and kept at 37 °C in water bath for 16 h.

### LC–MS conditions

After trypsin digestion, the samples were desalted and concentrated using ZipTip (Millipore, USA) and identified by mass spectrometry. A liquid chromatography-mass spectrometry (LC–MS) system was consisting of a Dionex Ultimate 3000 nano-LC system (nano UHPLC, Sunnyvale, CA, USA), connected to a linear quadruple ion trap orbitrap (LTQ Orbitrap XL) mass spectrometer (ThermoElectron, Bremen, Germany), and equipped with a nano electrospray ion source. For LC separation, an AcclaimPepMap 100 column (C18, 3 μm, 100 Å) (Dionex, Sunnyvale, CA, USA) capillary with a 15 cm bed length was used with a flow rate of 300 μL/min. Two solvents, A (0.1% formic acid) and B (aqueous 80% acetonitrile in 0.08% formic acid) were used to elute the peptides from the nano column. The gradient went from 5 to 40% B in 32 min and from 40 to 95% B in 1 min, with a total run time of 60 min. The flow rate was 300 μL/min and sample size was 5 μL. The mass spectrometer was operated in the data-dependent mode so as to automatically switch between Orbitrap-MS and LTQ-MS/MS acquisition. Electrospray voltage and the temperature of the ion transfer capillary were 2.2 kV and 200 °C, respectively. Survey full scan MS spectra (from m/z 350–1800) was acquired in the orbitrap with a resolution of 60,000 at m/z 400, allowing the sequential isolation of the top ten signal intensity ions for collision-induced dissociation at a collision energy of 35 V. A dynamic exclusion mode was enabled to exclude the previously selected ions during the repeated cycle of 60 s. The external mass calibration of the orbitrap was performed every 3 days to ensure a working mass accuracy <5 ppm.

### Data analysis

The protein profiles obtained from MS/MS spectra were compared to the watermelon proteins of the Universal Protein Resource database, (UniProt) (http://www.uniprot.org/datebase/identifier.format) using Maxquant software.

For protein identification, all criteria were set as follows: the comparison of the false discovery rate is 1%. Mass spectrometry precision parent ion was 10 ppm and fragment ion was 0.8 Da. The whole peptide fragment allowed two miss cut sit. The charge states of peptides were set to +2, +3, and +4. The confident protein identification involved at least two unique peptides. Ion score or expected was cut off less than 0.05 (with 95% confidence) and significance threshold p < 0.05 (with 95% confidence). The LFQ score of proteins with over 1.5-fold changes between any two samples and p value less than 0.05 were identified as differentially expressed proteins. The biological process and functions of identified proteins were according to the annotations of the Protein Information Resource (PIR) (http://pir.georgetown.edu/) database. Gene Ontology (GO) functional annotation analysis was used to analyze the expression level of all identified proteins. The GO analysis can describe the protein function and the relationship between different proteins precisely, which is widely used in protein function annotation (Berardini et al. 2004). In this study, gene ontology (GO) enrichment, kyoto encyclopedia of genes and genomes (KEGG) pathway analysis was established using a multi-omics data analysis tool, OmicsBean (http://www.omicsbean.cn).

## Results

### Colonization of strain SQR-21 on the watermelon roots

**Fig. 2:**
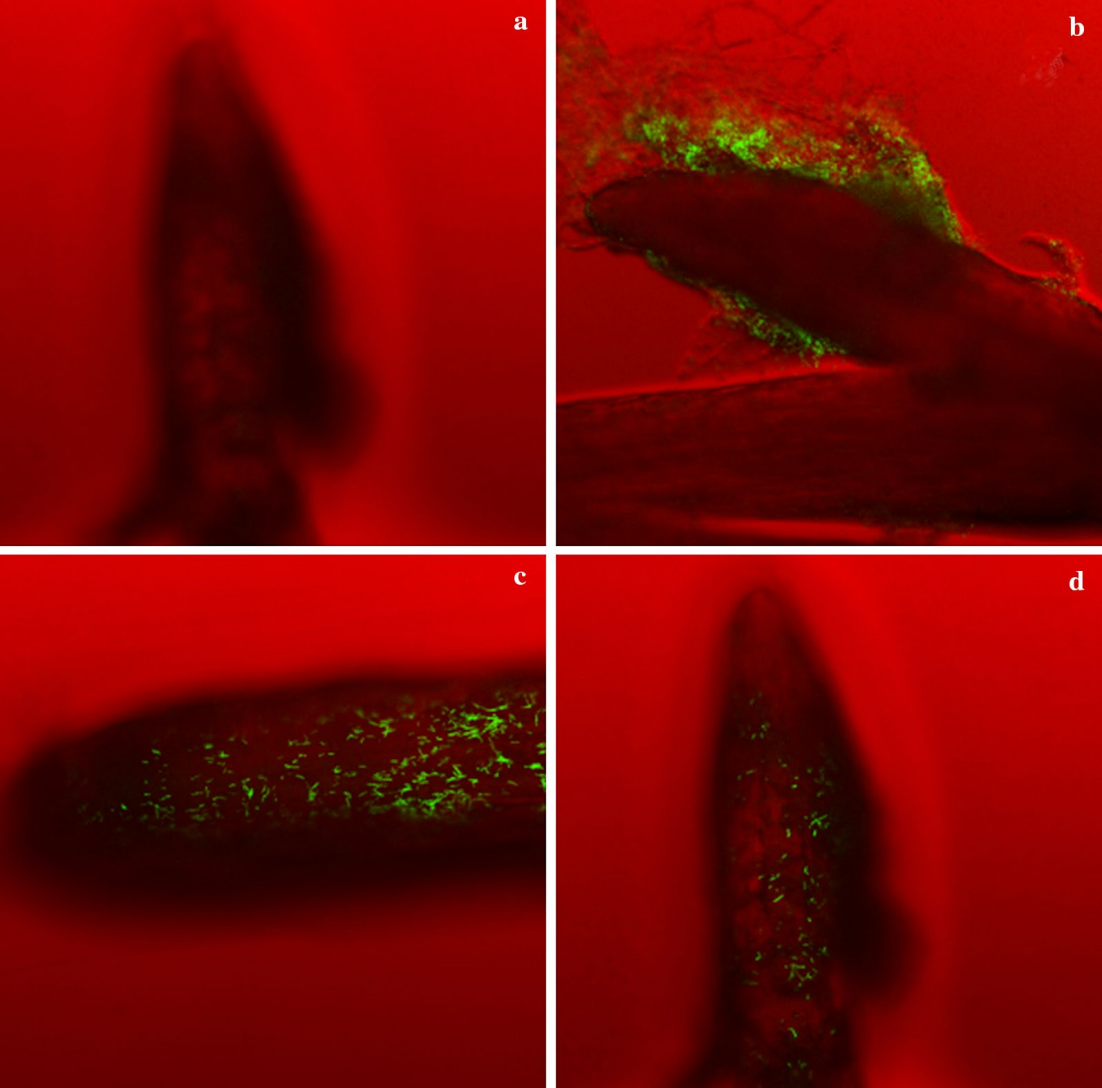
Qualitative analysis of SQR-21-*gfp* cells colonized on watermelon roots. **a** Without inoculation of SQR-21-*gfp* to the watermelon roots as a control; after **b** 1, **c** 3, and **d** 5 days, SQR-21-*gfp* colonized on the watermelon roots

In the hydroponic culture system, 1 day after inoculation, the concentration of SQR-21 cells colonized on the watermelon roots was 4.5 × 10^6^ cfu/g fresh roots. Three days later, the concentration of colonized SQR-21 cells was 1.4 × 10^5^ cfu/g fresh roots and the concentration was reached to 4.8 × 10^4^ cfu/g in fresh roots after 5 days. In fluorescence micrographs, the colonization of SQR-21 cells could also be seen clearly on watermelon roots (Fig. 2). The results indicated that strain SQR-21 colonized the roots of watermelon successfully.

### Protein identification and quantification

**Fig. 3:**
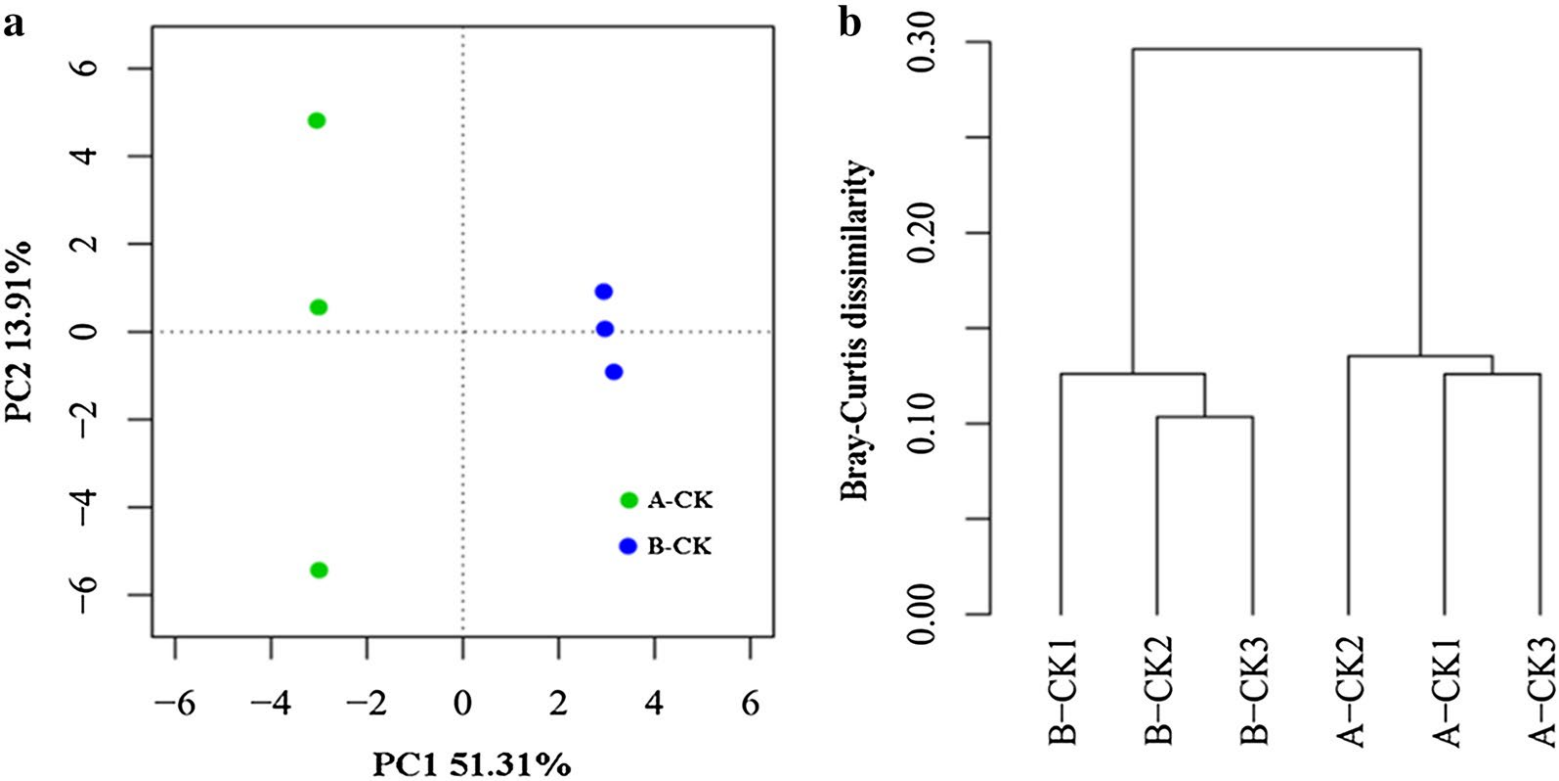
**a** Principal component analyses (PCA) and **b** cluster analysis of each sample by Ward method. *B-CK* treatment of inoculated SQR-21 without bacteria side, *A-CK* no bacteria inoculated as control

Protein identification and quantification were established based on Maxquant software with signal strength to finish proteomics Label free Quantitation Technique. A total of 623 kinds of proteins were identified. Control sample contained 435 proteins. For the treatment samples, no SQR-21 cells inoculated side (B-CK) had 447 proteins. The principal component analysis and cluster analysis showed significant differences between the treatment and control (Fig. 3). To confirm differentially expressed proteins between the treatment (B-CK) and control (A-CK), in the present study, only proteins that were identified with more than 1.5-fold changes and *p* value ≤0.05 between two samples were considered as differentially expressed proteins. A total of 119 differentially expressed proteins including 57 and 62 proteins were up-regulated and down-regulated respectively when compared treatment with the control. The information of all differentially expressed proteins was listed in the Additional file 1: Table S1.

### Gene Ontology enrichment analysis for differentially expressed proteins

**Fig. 4:**
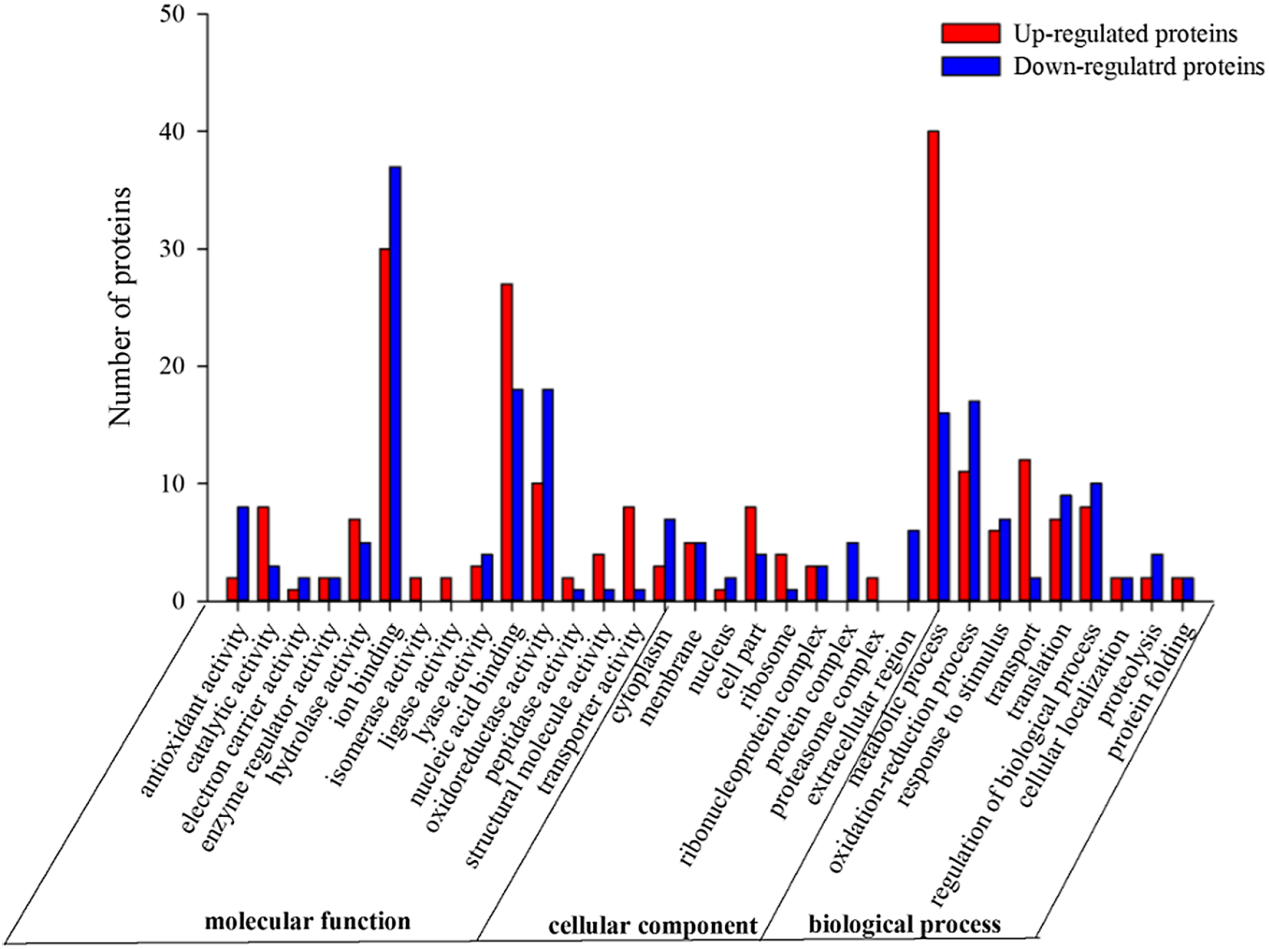
GO Ontology analysis of differentially expressed proteins of compared treatment (B-CK) with the control (A-CK). The proteins were categorized according to the annotation of GO, and the number of each category is displayed based on biological process, cellular components, and molecular functions. *Red color bars* indicate up-regulated and *blue color bars* indicate down-regulated proteins

The GO enriched analysis results cover a wide range of cellular components, biological processes and molecular functions. For biological process category, 40 up-regulated proteins were involved in metabolic process, including organic acid metabolic process, nucleotide and nucleoside metabolic process, while 12 proteins were involved in transport process. Among the down-regulated proteins, 17 proteins involved in oxidation–reduction process, while 16 proteins involved in metabolic process were found. It was also noticed that only two down-regulated proteins involved in transport process were identified. In the cellular component category, several key cellular components were identified, such as cell part, membrane and ribosome. For molecular function, all the differentially expressed proteins were categorized into 14 class functions. The highest occurrence frequency of the function classifications was ion binding, oxidoreductase activity, nucleic acid binding, and transporter activity (Fig. 4). Particularly, the number of up-regulated proteins involved in transporter activity and nucleic acid binding were more than down-regulated proteins.

### Cluster of orthologous groups classed analysis for differentially expressed proteins

**Fig. 5:**
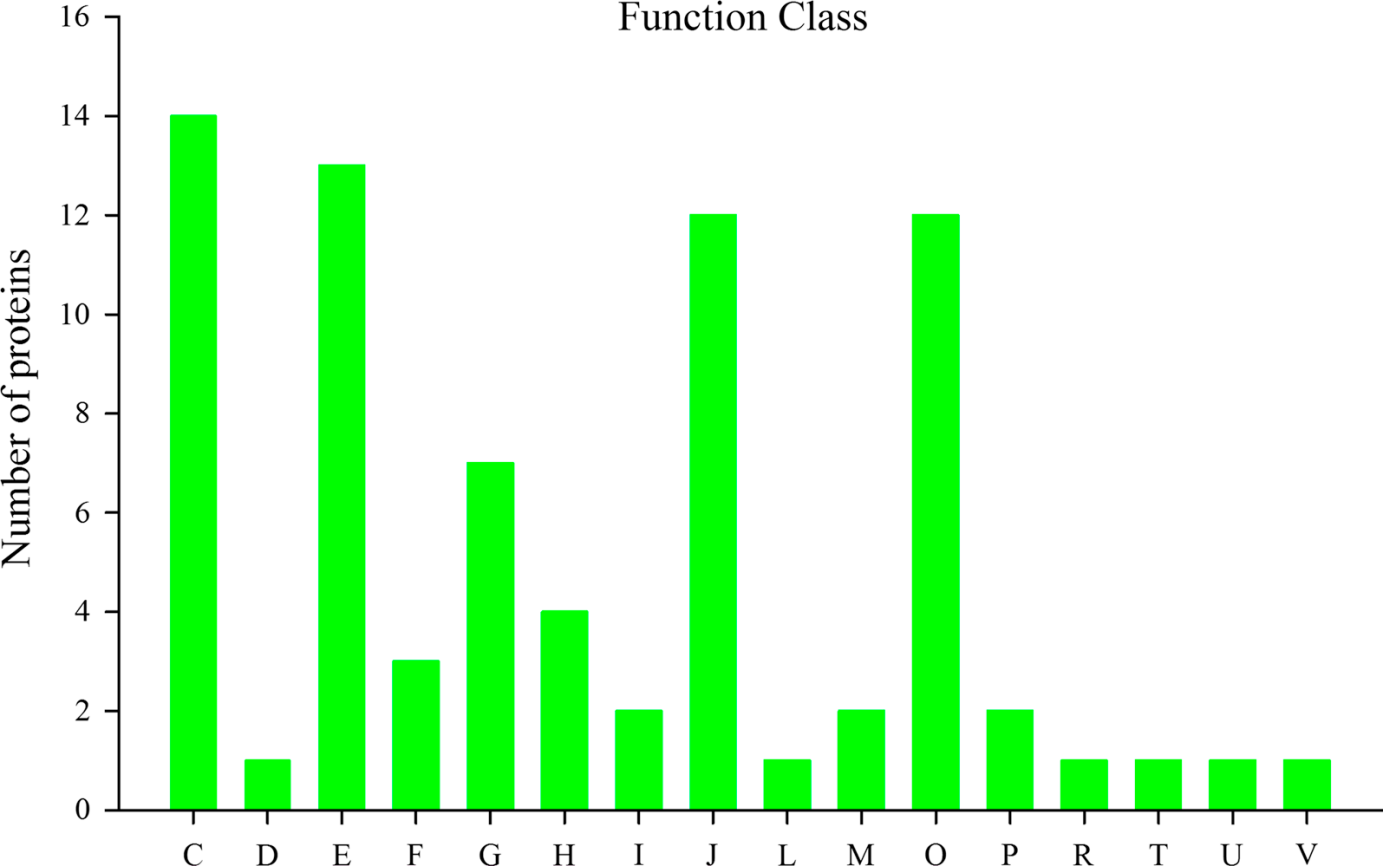
Functional classification of proteins by COG. *C* Energy production and conversion. *D* Cell division and chromosome partitioning. *E* Amino acid transport and metabolism Coenzyme. *F* Nucleotide transport and metabolism. *G* Carbohydrate transport and metabolism. *H* Coenzyme transport and metabolism. *I* Lipid transport and metabolism. *J* Translation, ribosomal structure and biogenesis. *L* Replication, recombination and repair. *M* Cell wall/membrane/envelope biogenesis. *O* Posttranslational modification, protein turnover, chaperones. *P* Inorganic ion transport and metabolism. *R* General function prediction only. *T* Signal transduction mechanisms. *U* Intracellular trafficking, secretion, and vesicular transport. *V* Defense mechanisms

Cluster of orthologous groups (COG) of proteins is the database for protein orthologous classification. Through comparing identified proteins with COG database to predict function of proteins, the results showed that differentially expressed proteins were classified into 16 clusters (Fig. 5). The highest frequencies of occurrence of the functional classification were energy production and conversion (14 proteins), followed by amino acid transport and metabolism (13 proteins), post-translational modification (12 proteins), and translation ribosomal structure and biogenesis (12 proteins). In addition, there also have 7 proteins involved in carbohydrate transport and metabolism, 4 proteins involved in coenzyme transport and metabolism. Indicated these functional classifications proteins were more abundant when SQR-21 colonized the watermelon roots.

### Pathway enrichment analysis for differentially proteins expression

**Fig. 6:**
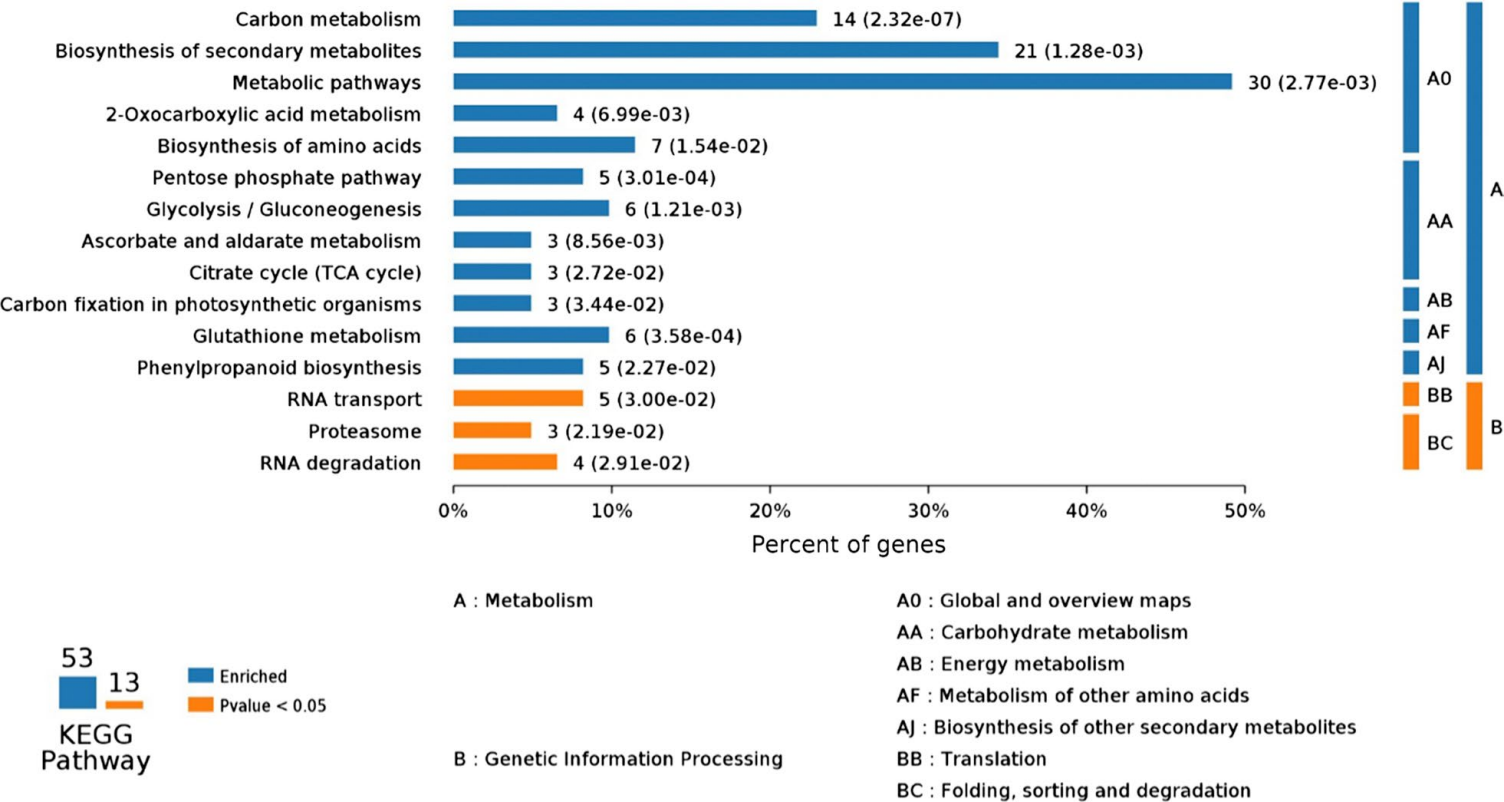
Compared treatment (B-CK) with the control (A-CK), the differentially expressed proteins KEGG pathway enrichment. The number of involved proteins in a specific pathway and corresponding p value are shown on the *right side of column*

Kyoto encyclopedia of genes and genomes (KEGG) pathway analysis was performed to evaluate the effect of SQR-21 on watermelon roots metabolic pathways (Fig. 6). After the application of KEGG blast search, differentially expressed proteins mapped 53 KEGG pathways and 13 of them significantly enriched, with the situation of some proteins involved in more than one pathway, including metabolic pathway contain COX6A, VHA-B2, PER47 and PER54 etc.; biosynthesis of the secondary metabolites contain ADH2, At3g58610, CSY4, At3g08590, PER54, PER72 and At4g27270 etc.; carbon metabolism; contain A0A0A0KKC3, ADH2, GDH1, At1g64190, NADP-ME3 and A0A0A0LEK8 etc.; biosynthesis of amino acids contain At3g58610, CSY4, At3g08590, ENO1, At4g17830 and CICDH; carbon fixation in photosynthetic organisms contain At2g36460, MDH1 and NADP-ME3; glutathione metabolism contain GSTF7, GSTU22, GSTF10, At1g64190, CICDH and ACG12; glycolsis/gluconeogenesis contain ADH2, At3g08590, At2g36460, PGI1 and ENO1; RNA degradation contain PAB2, ENO1. RH6 and CPN60B1; ascorbate and aldarate metabolism contain UGD2, At3g52880 and At5g21105; pentose phosphate pathway contain At2g36460, PGI1, At1g64190 and ACG12; 2-Oxocarboxylic acid metabolism contain At3g58610, CSY4, CICDH and At4g17830; phenylpropanoid biosynthesis contain PER54, PER72, PER47 and PER55; Citrate cycle (TCA cycle) contain CSY4, MDH1 and CICDH; Proteasome contain RPN3, ARPN1A and ARPN2B (Additional file 1: Table S1).

## Discussion

Proteomics is a new developing technique to study high throughput and systematize bioinformatics followed by genomics. Proteins carry out most biological functions, so proteomics investigates how proteins work in the cell within specific structures or complexes (Anderson et al. 2000; Dutt and Lee 2000). In this study, we extracted watermelon root proteins and used label free quantitative analysis to investigate the regulation of SQR-21 and watermelon interactions. The results showed that watermelon root protein profile was extremely changed after inoculation with SQR-21, especially those involved in physiological functions and pathways like signal transduction, cell motility, degradation of plant polysaccharides, biocontrol, and detoxification signal transduction.

### Signal transduction

Plant growth and development is mainly controlled by the regulation of genetic information and environmental conditions. Genes determines the basic pattern of development, so plant growth is largely controlled by environmental stimulation or environmental information (Yoshito et al. 1991). ADP-ribosylation factor was detected up-regulated in the SQR-21 inoculated root samples, which is a kind of GTP-binding protein family. The GTP-binding protein mainly participates in biological process like cell communication, regulation of biological process and response to stimulus. G-protein through coupled receptors (GPCR) regulatory enzyme and ion channels of bio-membrane effective apparatus produce the intracellular signal to adjust specific cellular responses (Paduch et al. 2001). Signal-transducing GTPases in plants include small G-proteins, heterotrimeric G proteins, and potentially several unique types of GTP binding proteins that are not members of either of the aforementioned classes (Assmann 2002). In this study, inoculation of SQR-21 the expression of GTP binding proteins has been up-regulated, and the communication between plant roots and the environment was improved, which promoted watermelon roots growth and defense mechanism. Phospholipase D (PLD), a down-regulated protein in the SQR-21 treated samples, is not only an important phospholipid hydrolase, also a transmembrane signal transduction enzyme (Wang 1999). The process that phospholipase hydrolyze phospholipids involved is one of plant membrane signal transduction pathway (Billah 1993). Phospholipids in under the action of phospholipid, hydrolyzed to fatty acid, IP3 (Inositol triphosphate), PA (phosphatidic acid), DAG (diacylglycerol) and acetyl choline. Recent studies have shown that these hydrolysates are second messenger, finished the process of cell response to external signal (Chapman 1998). Young et al. found that *Xanthomonas oryzae* infected to rice, PLD activity has been improved significantly, especially in disease-resistant variety (Young et al. 1996). In our study, inoculation of SQR-21, the expression of PLD has been down-regulated, showing a dissimilar response of induced plant resistance by pathogens.

### Transportation

The transportation system plays a key role in the communication and adaptation of plant with their environment. In this study, the inoculation of watermelon roots with SQR-21, the up-regulated protein like aspartate amino-transferase (AAT), which is located in cytoplasm, mitochondria and chloroplasts, ribosomes and golgi apparatus. AAT is the key enzyme in the nitrogen metabolic pathways, it catalytic grass phthalein acid and glutamic acid to produce aspartic acid and ketone glutaric acid (Givan 1980). In the process of the root absorption of ammonia, recycled use carbon skeleton, provide the precursor for the synthesis of nitrogen transfer objects (Rawstone et al. 1980). There were many researches showing that high temperature, low-nitrogen and transformation exogenous gene could affect the expression of the AAT, while microbial induced the AAT research has not been reported (Murooka et al. 2002; Zhou et al. 2009). Porin, an down-regulated proyein, which allowing small biomolecules like mono- and disaccharides, nucleosides, and amino acids for rapid diffusion across the outer membrane (Welte et al. 1995). Besides, some up-regulated proteins like eukaryotic translation initiation factor 3 subunit E, involved in nuclear transport, some uncharacterized protein (genename: RH6,VHA-B2) involved in nucleobase-containing compound transport, hydrogen ion transmembrane transport. All those protein are significant changed. The results showed that SQR-21 inoculated on watermelon roots could promote root amino acid, sugar, and nitrogen transport and maintain cell morphology to ensure the watermelon growth.

### Carbohydrate metabolic process

During the whole life of one cell, it needs energy to maintain various activities, as well as to keep the balance of life. Energy metabolism is important for the normal development of the life foundation. This two kinds of up-regulated proteins glucose-6-phosphate dehydrogenase (G6PDH) and 6-phosphogluconate dehydrogenase (6PGDH) are two key enzymes catalyzes the rate limiting step of the oxidative pentose-phosphate pathway, exhibiting an important role in the reduced form of nicotinamide adenine dinucleotide phosphate (NADPH) regeneration (Nicol et al. 2000). G6PDH and 6PGDH are not only play an important role for the growth of organisms,they provides the NADPH is involved in many reactions or cycle in cell, and provide reducing power for these reaction (Wakao and Benning 2005). Sindelar et al. (1999) found when tobacco leaf tissues infected by potato virus Y, the activity of G6PDH and 6PGDH in tobacco tissues was significantly enhanced. In this study this two proteins are significantly up regulated. Result show that, SQR-21 induced G6PDH and 6PGDH expression in the same way as virus did. Sucrose synthase is one of the widely exist in plants glycosyl transferase, also up regulated, which as a cytoplasmic enzyme in degrading sucrose and provides carbon for respiration and the synthesis of cell wall polysaccharides in plant cells (Zuzanna et al. 2007). UDP-glucose pyrophosphorylase was up-regulated protein in SQR-21 inoculated sample. UGPase presents an important activity in carbohydrate metabolism, catalyzing a reversible production of UDPG and pyrophosphate (PPi) from glucose-1-phosphate (Glc-1-P) and uridine triphosphate (UTP), another important function of UDPase is the synthesis of plant cell walls (Winter et al. 2000). Fan et al. showed that citrus sinensis infected by *Candidatus Liberibacter asiaticus* leading to soluble sugar accumulation. Soluble sugar not only a major way of energy transportation, but is life activity to use of carbohydrate direct form (Fan et al. 2010). In this study, compared to control, the SQR-21 inoculated root sample showed higher expression of proteins related to carbohydrate metabolism, which might increase the activity of pentose phosphate pathway and metabolism to release energy which ensured the plant growth, development and response to environmental stresses.

### Defense and response to stress

Plants are often exposed to natural and synthetic toxins such as heavy metals, allelochemicals, organic contaminants, and pesticides (Riechers et al. 2010). Cell wall can resist germs harm and adversity. Plant polysaccharides consist of plant cell wall polysaccharides (cellulose, hemicelluloses, and pectin) and storage polysaccharides including many different monomers associated with each other by a diversity of linkages (Vries and Visser 2001; Vries et al. 2011). In this study, we detected the protein caffeoyl-CoAO-methyltransferase (CCoAOMT) an up-regulated protein, which plays crucial roles in lignin biosynthesis. Lignin exists in plant secondary cell wall, increases the hardness and compressive strength of the plant, at the same time resist damage, metabolic stress and biotic and abiotic stresses (Boerjan et al. 2003; Vanholme et al. 2010). Glutathione S-transferase (GST) has been up-regulated. A large number of soluble GST are positioning in the cytoplasm, a small amount of GST in or outside the cell nucleus (Edwards et al. 2000). They are translation induced by many different environmental factors, and play important roles not only in many stress responses, but also in plant growth, development and secondary metabolism. Wagner et al. identified a new up-regulated protein family, glutathione S-transferases (GSTs), in *Arabidopsis* flowers and leaves by inoculation of *Fusarium sporotrichioides* and *Fusarium graminearum* to *Arabidopsis* flowers and leaves (Wagner et al. 2002). Glutamate decarboxylase (GAD) an intracellular enzyme that existed in the cytoplasm, was detected up-regulated in the treatment sample, combination with pyridoxal phosphate cofactors can catalyze glutamic acid into gamma-aminobutyric acid (GABA) and CO _2_. Plant GAD is regulated by Ca^2+^ levels since it has a calmodulin-binding site in the C-terminal region, which is found only in the plant enzymes (Chen et al. 1995; Hiroshi 2000). When plants faced to different stresses, such as hypoxia, mechanical stress, plant hormones, cold shock, heat shock, and water stress, GAD are activated by the increased intracellular H^+^ and Ca^2+^ levels to accumulate GABA, which plays an important role in the pH control, nitrogen storage, plant development and resistance processes (Pedro et al. 1995). Another up-regulated protein ubiquitin-activating enzyme E1 was a key enzyme in proteasome pathway. Protein degradation through the ubiquitin–proteasome system is the major pathway of non-lysosomal proteolysis of intracellular proteins Aberration of this system leads to the dysregulation of cellular homeostasis and the development of multiple diseases (Hershko et al. 1983). This is an important way to regulating the protein activity (Pickart 2001; Smalle and Vierst 2004). In conclusion, the differently expressed proteins mentioned above could induce plant cell wall lignifications, increase the strength of the plant cell wall, adjust the physiological activity of plants, and strengthen the resistance related compounds synthesis, which could protect watermelon roots from different stresses.

### Oxidation–reduction process

In plants, many secondary metabolites are toxic to cells, even for these cells produce these substances were also poisonous (Marrs et al. 1995). The following two up-regulated proteins thioredoxin peroxidase (TPX) and ascorbate peroxidase (APX) involved in plant detoxification pathway. TPX belongs to peroxiredoxin family (Circu and Aw 2010), which can participate in kinds of reaction of clear peroxide to improve the resistance of plants (Woo et al. 2010). APX is scavenger of H_2_O_2_, we know that H_2_O_2_ belongs to reactive oxygen species (ROS) which could use Haber–Weiss reaction to generate high active radical and destroy cell death. APX using ascorbic acid salt as its special electron donor to restore H_2_O_2_ for H_2_O, and accompany single dehydrogenation ascorbic acid (DHA) produced. Protect the cells from the toxic effects of reactive oxygen species and plays a vital function in the process of ROS (Teixeira et al. 2004; Ishikawa and Shigeoka 2008). Many studies about peroxidases in plants have been reported to response to the pathogen infection, such as *Arabidopsis* infected by *Fusarium*, rice infected by *Pyricularia grisea* and strawberry leaves inoculated with *Colletotrichum* (Chivasa et al. 2006; Fang et al. 2012). Our results supported by these publications. Results in this study demonstrated that involved in oxidation–reduction process and detoxification related proteins more expression after the inoculation of SQR-21, by clear peroxide, ROS and destructive protein to enhanced watermelon stress tolerance.

The inoculation of *P. polymyxa* SQR-21 changed the expression of protein in watermelon roots enormously. Further analysis indicated that the SQR-21 strain could increase the abundance of potentially beneficial proteins involved in signal transduction, carbohydrate metabolic and degradation of plant metabolism, transport, biocontrol and detoxification. These results suggested that PGPR strain *P. polymyxa* SQR-21 could potentially promote host plant growth by altering root protein composition. This study would help to better understand the plant–microbe interaction mechanisms involved in plant growth promotion and protection against pathogens which will be helpful to promote the application of PGPR strains in agricultural production.

## Additional file

**Additional file 1: Table S1.** Differentially expressed proteins identified in watermelon roots after inoculated with SQR-21.

## Abbreviations

SQR-21: *Paenibacillus polymyxa* SQR-21
LC-MS: liquid chromatography-mass spectrometer
PGPR: plant growth promoting rhizobacteria
ACC: 1-aminocyclopropane-1-carboxylate deaminase
VOCs: volatile organic compounds
QS: quorum sensing
GBR-1: *P. polymyxa* GBR-1
HKA-15: *P. polymyxa* HKA-15
iTRAQ: isobaric tags for relative and absolute quantification
CLSM: confocal laser scanning microscope
UA: urea aqueous solution
DTT: DL-dithiothreitol
IAM: iodacetamide
Da: dalton
PIR: protein information resource
LFQ: label-free quantitation
GO: gene ontology
KEGG: kyoto encyclopedia of genes and genomes
COG: cluster of orthologous groups
ADP: adenosine diphosphate
GPCR: G-protein through coupled receptors
G-proteins: guanosine triphosphate protein
GTP: guanosine triphosphate
PLD: phospholipase D
IP3: inositol triphosphate
PA: phosphatidic acid
DAG: diacylglycerol
AAT: aspartate amino-transferase
G6PDH: glucose-6-phosphate dehydrogenase
6PGDH: 6-phosphogluconate dehydrogenase
NADPH: nicotinamide adenine dinucleotide phosphate
UDPG: UDP-glucose pyrophosphorylase
PPi: pyrophosphate
Glc-1-P: glucose-1-phosphate
UTP: uridine triphosphate
CCoAOMT: caffeoyl-CoA 3-O-methyltransferase
GST: glutathione S-transferase
GAD: glutamate decarboxylase
GABA: gamma-aminobutyric acid
TPX: thioredoxin peroxidase
APX: ascorbate peroxidase
ROS: reactive oxygen species
DHA: dehydrogenation ascorbic acid
2-DE: two-dimensional electrophoresis
