# Supplementary material for: PGPR strain Paenibacillus polymyxa SQR-21 potentially benefits watermelon growth by re-shaping root protein expression
Source: AMB Express. 2017 May 25;7:104. doi: 10.1186/s13568-017-0403-4 (PMC5445060; doi:10.1186/s13568-017-0403-4)
Supplement: Supplementary file 1 — Additional file 1: Table S1. Differentially expressed proteins identified in watermelon roots after inoculated with SQR-21. [file 13568_2017_403_MOESM1_ESM.docx]

**AMB Express**

**PGPR strain** ***Paenibacillus polymyxa* SQR-21 potentially benefits watermelon growth by re-shaping root protein expression**

Yaoyao E^a^, Jun Yuan^a^, Fang Yang, Lei Wang, Jinghua Ma, Jing Li, Xiaowei Pu, Waseem Raza, Qiwei Huang^[[1]](#footnote-1)^*, Qirong Shen

Jiangsu Provincial Key Lab of Organic Solid Waste Utilization and Jiangsu Collaborative Innovation Center for Organic Solid Waste Utilization; Nanjing Agricultural University, Nanjing, 210095, China.

^a^Both authors contributed equally to this paper

**Running title: PGPR benefits plants by altering root protein expression**

*Corresponding author: Prof. Qiwei Huang, College of Resources and Environmental Sciences, Nanjing Agricultural University, 210095, Nanjing, Jiangsu Province, P. R. of China

E-mail address: qwhuang@njau.edu.cn, Tel: (86)025-84396824; Fax: (86)025-84396291

Table S1. Differentially expressed proteins identified in watermelon roots after inoculated with SQR-21.

| **Accession** | **Protein Name** | **Gene name** | **Peptides** | **coverage**  **[%]** | **Biological Process** | **Fold Change** | **p-value** |
| --- | --- | --- | --- | --- | --- | --- | --- |
| **Function 1. Transport** | | | | | | | |
| Q8RW69 | Isocitrate dehydrogenase [NADP] | CICDH | 14 | 35.9 | regulation of transport | 1.58 | 0.00193 |
| A0A0A0LC61 | Porin | A0A0A0LC61 | 5 | 23.9 | transport | 0.55 | 0.002089 |
| Q2Q0V7 | Patellin 1 | Q2Q0V7 | 8 | 18.9 | transport | 3.33 | 0.009722 |
| E5GB78 | Adenine nucleotide translocator | E5GB78 | 6 | 18.2 | transport | 1.79 | 0.025479 |
| A0A0A0KRL4 | Uncharacterized protein | A0A0A0KRL4 | 3 | 19.6 | hydrogen transport | 0.62 | 0.000542 |
| A0A0A0LCA0 | Uncharacterized protein | A0A0A0LCA0 | 4 | 11.9 | hydrogen transport | 1.90 | 0.011575 |
| A0A0A0K4R0 | Uncharacterized protein | VHA-B2 | 13 | 33.5 | hydrogen ion transmembrane transport;nucleoside triphosphate metabolic process | 2.21 | 0.000602 |
| A0A0A0LSH7 | Uncharacterized protein | RH6 | 10 | 32.1 | nucleobase-containing compound transport | 2.80 | 0.00591 |
| A0A0A0L0F9 | Eukaryotic translation initiation factor 3 subunit E | TIF3E1 | 6 | 17.8 | nuclear transport | 0.33 | 1.42E-05 |
| **Function 2.oxidation-reduction process** | | | | | | | |
| A0A0A0LG46 | Uncharacterized protein | A0A0A0LG46 | 21 | 36.1 | oxidation-reduction process;response to stimulus | 0.55 | 0.008859 |
| A0A0A0K199 | Uncharacterized protein | A0A0A0K199 | 4 | 20.6 | oxidation-reduction process;response to stimulus | 0.39 | 0.000108 |
| A0A0A0L6H3 | Uncharacterized protein | A0A0A0L6H3 | 8 | 16 | oxidation-reduction process | 1.57 | 0.03315 |
| A0A0A0KM88 | Uncharacterized protein | A0A0A0KM88 | 3 | 6.6 | oxidation-reduction process | 0.33 | 0.000878 |
| D3JWE4 | Thioredoxin peroxidase | D3JWE4 | 3 | 59.3 | oxidation-reduction process | 2.98 | 0.024261 |
| E2S0A6 | Cytosolic alkenal/one oxidoreductase | E2S0A6 | 5 | 19.1 | oxidation-reduction process | 2.83 | 0.002856 |
| **Function 3.response to stimulus** | | | | | | | |
| A0A0A0L0X1 | Thioredoxin reductase | NTR2 | 5 | 23.7 | response to oxygen radical | 0.49 | 2.39E-05 |
| Q9SBL8 | Sucrose synthase | SUS4 | 23 | 36 | response to biotic stimulus | 1.80 | 0.043321 |
| A0A0A0M2C3 | Uncharacterized protein | CPN60B1 | 5 | 12.3 | response to abiotic stimulus | 2.32 | 0.008268 |
| A0A0A0L2A9 | Peroxidase | PER47 | 3 | 14.2 | response to inorganic substance | 0.39 | 0.004715 |
| A0A0A0KYN6 | Glutamate dehydrogenase | GDH1 | 6 | 21.7 | response to metal ion | 1.77 | 0.005795 |
| A0A0A0K3C4 | Uncharacterized protein | RPN1A | 7 | 10.7 | response to chemical | 1.80 | 0.004606 |
| A0A0A0LGI3 | Fructose-bisphosphate aldolase | At2g36460 | 6 | 23.2 | response to chemical | 0.36 | 0.004164 |
| A0A059VCH7 | Peroxidase | A0A059VCH7 | 4 | 18.6 | response to stimulus | 0.64 | 0.023927 |
| **Function 4.protein structure change** | | | | | | | |
| A0A0A0LTY6 | Calnexin | CNX1 | 8 | 15.2 | protein folding | 0.52 | 0.000517 |
| A0A0A0LZU0 | Uncharacterized protein | A0A0A0LZU0 | 6 | 13.3 | protein folding | 1.89 | 0.01546 |
| A0A0A0LFZ8 | Uncharacterized protein | A0A0A0LFZ8 | 7 | 17.9 | protein folding | 0.41 | 0.001053 |
| A0A0A0KSN9 | Uncharacterized protein | A0A0A0KSN9 | 2 | 4.1 | protein folding | 1.66 | 0.041846 |
| A0A0A0KZT6 | Heat shock protein | A0A0A0KZT6 | 8 | 15.3 | protein folding | 0.59 | 0.015337 |
| A0A0A0LNA7 | Guanosine nucleotide diphosphate dissociation inhibitor | GDI2 | 10 | 29.1 | protein localization | 5.22 | 0.002918 |
| A0A0A0LV78 | Uncharacterized protein | RANBP1C | 4 | 31.5 | protein localization to nucleus | 0.38 | 0.000236 |
| A0A0A0LNS8 | Ribonucleoprotein | CP31A | 2 | 6.9 | protein localization | 0.41 | 0.010564 |
| A0A0A0KE12 | Ubiquitin-activating enzyme E1 | A0A0A0KE12 | 10 | 11.5 | cellular protein modification process | 1.56 | 0.039385 |
| A0A0A0L1P5 | Uncharacterized protein | AGO4 | 6 | 7.4 | protein methylation | 1.99 | 0.009932 |
| **Function 5.biosynthetic process** | | | | | | | |
| A0A0A0LMD3 | Uncharacterized protein | OPR1 | 5 | 19.1 | fatty acid biosynthetic process | 0.59 | 0.048552 |
| A0A0A0L2P8 | Uncharacterized protein | GSTF10 | 3 | 11.6 | organonitrogen compound biosynthetic process | 0.61 | 0.038962 |
| A0A0A0K7P9 | Eukaryotic translation initiation factor 3 subunit F | TIF3F1 | 3 | 13.7 | organonitrogen compound biosynthetic process\| | 0.29 | 0.000195 |
| Q9AXQ2 | Mitochondrial processing peptidase beta subunit | At3g02090 | 6 | 16.7 | small molecule biosynthetic process | 3.99 | 0.001183 |
| Q9MB49 | DIP-1 | At4g17830 | 9 | 32.9 | small molecule biosynthetic process | 0.61 | 0.041082 |
| A0A0A0LRI8 | Uncharacterized protein | At2g18110 | 8 | 27.9 | cellular biosynthetic process | 0.48 | 0.013608 |
| A0A0A0K909 | Elongation factor Tu | A2 | 3 | 10 | cellular biosynthetic process | 1.81 | 0.01922 |
| Q9ZR60 | L-ascorbate oxidase | At5g21105 | 7 | 22.7 | sphingolipid biosynthetic process | 0.32 | 0.00846 |
| **Function 6.cell development** | | | | | | | |
| A0A0A0LPK6 | Uncharacterized protein | ENO1 | 6 | 22 | cell development | 0.35 | 0.000252 |
| A0A0A0LW91 | Malic enzyme | NADP-ME3 | 14 | 33.5 | developmental growth | 3.43 | 0.00059 |
| A0A0A0KZ30 | UDP-glucose 6-dehydrogenase | UGD2 | 10 | 31 | root system development | 1.82 | 0.004694 |
| A0A0A0LK91 | Alpha-galactosidase | AGAL3 | 3 | 7.8 | root system development | 2.78 | 0.001512 |
| A0A0A0LNE3 | Adenosylhomocysteinase | SAHH1 | 13 | 40.4 | root system development | 1.51 | 0.026564 |
| **Function 7.regulation process** | | | | | | | |
| A0A0A0KXT6 | Uncharacterized protein | A0A0A0KXT6 | 3 | 8 | regulation of biological process | 0.52 | 0.045406 |
| A0A0A0LUQ2 | Protein disulfide-isomerase | A0A0A0LUQ2 | 6 | 14.9 | regulation of biological process | 0.60 | 0.022096 |
| A0A0A0LRW2 | Glucose-6-phosphate isomerase | PGI1 | 11 | 21 | regulation of metabolic process | 1.59 | 0.031548 |
| A0A0A0LLF4 | Glucose-6-phosphate isomerase | PGI1 | 5 | 14.3 | regulation of metabolic process | 2.29 | 0.003916 |
| A0A0A0K1C2 | Uncharacterized protein | RPN3A | 5 | 13.7 | regulation of primary metabolic process | 2.62 | 0.022068 |
| A0A0A0LLW6 | Uncharacterized protein | RPN2B | 5 | 37.1 | regulation of primary metabolic process | 0.16 | 3.3E-05 |
| A0A0A0L325 | Minor allergen Alt a | At4g27270 | 3 | 17.7 | negative regulation of metabolic process | 2.17 | 0.001747 |
| A0A0A0L989 | Polyadenylate-binding protein | PAB2 | 6 | 8.1 | regulation of primary metabolic process | 0.32 | 0.041081 |
| A0A0A0LTB3 | Eukaryotic translation initiation factor 5A | ELF5A-1 | 4 | 21.9 | positive regulation of cellular amide metabolic process | 0.45 | 0.009986 |
| **Function 8.translation** | | | | | | | |
| A0A0A0LDW3 | Uncharacterized protein | A0A0A0LDW3 | 6 | 20.2 | translation | 0.39 | 0.014026 |
| A0A0A0KSN1 | Uncharacterized protein | A0A0A0KSN1 | 3 | 17.3 | translation | 2.31 | 0.001052 |
| A0A0A0KDI4 | Uncharacterized protein | A0A0A0KDI4 | 9 | 20.4 | translation | 0.39 | 0.030791 |
| A0A0A0K500 | Uncharacterized protein | A0A0A0K500 | 5 | 36.4 | translation | 0.65 | 0.030072 |
| Q6UNT2 | 60S ribosomal protein L5 | Q6UNT2 | 3 | 9.3 | translation | 3.07 | 2.37E-05 |
| **Function 9.metabolic process** | | | | | | | |
| A0A0A0K2N1 | Caffeoyl-CoA O-methyltransferase | A0A0A0K2N1 | 4 | 22.2 | metabolic process | 2.05 | 0.008843 |
| H6WX41 | Alkaline alpha galactosidase 3 | H6WX41 | 7 | 11.2 | metabolic process | 1.93 | 0.030087 |
| A0A0A0KUH2 | Uncharacterized protein | RAD23C | 5 | 16.7 | primary metabolic process；nucleic acid metabolic process | 0.37 | 0.000114 |
| A0A0A0LHT8 | Uncharacterized protein | At2g34970 | 5 | 16.1 | primary metabolic process | 0.60 | 0.026017 |
| A0A0A0LW19 | Uncharacterized protein | At5g08100 | 4 | 20.4 | primary metabolic process | 0.57 | 0.002293 |
| E5GBI8 | ADP-ribosylation factor | T17J13.250 | 9 | 60.4 | primary metabolic process | 2.73 | 0.000263 |
| A0A0A0L3I0 | Uncharacterized protein | COX6A | 2 | 15.6 | single-organism process | 0.43 | 0.056291 |
| A0A0A0K4D9 | Uncharacterized protein | T22F8.130 | 5 | 24 | single-organism metabolic process | 2.68 | 0.000578 |
| A0A0A0LGL9 | Uncharacterized protein | At3g52880 | 8 | 24.7 | single-organism metabolic process | 0.34 | 0.002612 |
| A0A097BU00 | Alcohol dehydrogenase | ADH2 | 8 | 28.9 | single-organism metabolic process | 0.28 | 0.000361 |
| A0A0A0KVG8 | Uncharacterized protein | GSTU22 | 3 | 11.7 | single-organism catabolic process | 2.06 | 0.004068 |
| A0A0A0LT08 | Peroxidase | PER55 | 3 | 16.4 | single-organism catabolic process | 1.50 | 0.005804 |
| A0A0A0KZ63 | Peroxidase | PER54 | 6 | 23.4 | single-organism catabolic process | 0.21 | 5.33E-05 |
| A0A0A0KWW5 | Peroxidase | PER54 | 2 | 9.9 | single-organism catabolic process | 0.39 | 0.001032 |
| A0A0A0KKC3 | Threonine dehydratase | A0A0A0KKC3 | 2 | 4.7 | cellular amino acid metabolic process | 0.45 | 0.008089 |
| A0A0A0KHC3 | Threonine dehydratase | A0A0A0KHC3 | 7 | 15.3 | cellular amino acid metabolic process | 0.53 | 0.006764 |
| A0A0A0LEZ3 | Methionine synthase | A0A0A0LEZ3 | 14 | 22.9 | cellular amino acid metabolic process | 7.41 | 0.017546 |
| A0A0A0KPY1 | Glutamate decarboxylase | A0A0A0KPY1 | 5 | 16.2 | cellular amino acid metabolic process | 2.81 | 0.001854 |
| A0A0A0LEK8 | Aspartate aminotransferase | A0A0A0LEK8 | 7 | 15.9 | cellular amino acid metabolic process | 2.38 | 0.000677 |
| A0A0A0KSV4 | Uncharacterized protein | A0A0A0KSV4 | 7 | 15.6 | carbohydrate metabolic process | 1.97 | 0.002059 |
| A0A0A0KIK3 | Uncharacterized protein | A0A0A0KIK3 | 14 | 41.9 | carbohydrate metabolic process | 0.53 | 0.000975 |
| A0A0A0LW50 | Uncharacterized protein | A0A0A0LW50 | 1 | 2.9 | carbohydrate metabolic process | 0.59 | 0.027947 |
| A0A0A0KQQ5 | Uncharacterized protein | A0A0A0KQQ5 | 7 | 30.2 | carbohydrate metabolic process | 1.55 | 0.039306 |
| G9BSA5 | Beta-glucosidase | G9BSA5 | 2 | 8.6 | carbohydrate metabolic process | 0.31 | 0.000493 |
| A0A0A0KT44 | Alpha-galactosidase | A0A0A0KT44 | 5 | 19.5 | metabolic process；carbohydrate metabolic process | 0.55 | 0.003565 |
| Q8LK75 | Acid invertase | Q8LK75 | 6 | 13.3 | metabolic process；carbohydrate metabolic process | 0.58 | 0.036153 |
| A0A0A0LR54 | Uncharacterized protein | ADK1 | 6 | 36.4 | purine ribonucleoside monophosphate metabolic process | 0.60 | 0.000193 |
| A0A0A0LII5 | Malate dehydrogenase | MDH1 | 7 | 26.4 | purine ribonucleoside monophosphate metabolic process | 1.53 | 0.044321 |
| A0A0A0K5T7 | Ketol-acid reductoisomerase | At3g58610 | 4 | 9.9 | purine ribonucleoside monophosphate metabolic process;nucleotide biosynthetic process | 2.76 | 0.003457 |
| A0A0A0LPD0 | Cysteine protease | RD19A | 3 | 13.5 | purine ribonucleoside monophosphate metabolic process | 0.49 | 1.73E-05 |
| A0A0A0K6P2 | Citrate synthase | CSY4 | 7 | 22.5 | ribonucleoside diphosphate metabolic process | 1.76 | 0.042321 |
| A0A0A0KZH0 | Peroxidase | PER72 | 5 | 23.1 | cellular catabolic process | 0.27 | 0.00027 |
| A0A0A0K5B8 | Glutathione S-transferase | GSTF7 | 6 | 36.3 | toxin catabolic process | 5.18 | 0.000273 |
| A0A0A0LIC6 | Methylenetetrahydrofolate reductase | MTHFR2 | 4 | 7.4 | coenzyme metabolic process | 1.90 | 0.002016 |
| R4I3D6 | Glucose-6-phosphate 1-dehydrogenase | ACG12 | 6 | 16.1 | NADP metabolic process | 1.53 | 0.036058 |
| B9VWD5 | 6-phosphogluconate dehydrogenase, decarboxylating | At1g64190 | 12 | 32.9 | NADP metabolic process | 2.03 | 0.001116 |
| Q2Q0A8 | Phospholipase D | Q2Q0A8 | 11 | 20.7 | lipid metabolic process | 0.63 | 0.010846 |
| J9Y1K3 | Phosphoenolpyruvate carboxylase protein | J9Y1K3 | 18 | 24.9 | organic acid metabolic process | 2.17 | 0.001619 |
| Q19TV8 | UDP-glucose pyrophosphorylase | Q19TV8 | 11 | 32.4 | metabolic process; phosphorus metabolic process | 2.41 | 0.01029 |
| A0A0A0KB09 | Uncharacterized protein | At1g51860 | 3 | 6.5 | cellular protein metabolic process | 1.56 | 0.021844 |

1. * To whom correspondence should be addressed: E-mail:[qwhuang@njau.edu.cn](mailto:qwhuang@njau.edu.cn) [↑](#footnote-ref-1)
